# Yttrium 90 Therapy: Is the Future Surgical?

**DOI:** 10.1007/s00270-020-02645-9

**Published:** 2020-09-24

**Authors:** Benjamin Garlipp

**Affiliations:** Otto von Guericke University, Leipziger Str. 44, 39120 Magdeburg, Germany

Surgery for malignant liver tumors has evolved greatly in the recent past, with the amount of functioning liver parenchyma remaining after resection—referred to as the future liver remnant (FLR)—now being the major determinant of resectability. Hence, the quest for methods to enhance the FLR in patients at risk for postoperative liver failure overcoming the known limitations of portal vein embolization (PVE) or portal vein ligation (PVL) has gained interest. Optimizing the PVE technique using a combination of polyvinyl alcohol particles and venous plugs or coils, the combination of transarterial embolization and portal venous embolization, liver venous deprivation (LVD) combining portal and hepatic vein embolization, as well as the ALPPS (Associating Liver Partition and Portal vein Ligation for Staged hepatectomy) procedure have all been studied in this context, with mixed results and arguments in favor of and against all of these methods.

In this issue of CVIR, Gordon et al. [1] describe PVE with ^90^Yttrium-labeled glass microspheres infused via the portal vein (Y90 PVE) in an experimental study using 22 Sprague–Dawley rats. Animals were assigned to one of five cohorts receiving very high, high, medium, and low-dose Y90 PVE or no treatment, respectively. Seventeen rats survived until necropsy at 12 weeks following Y90 PVE. Of the surviving 13 rats in the treated group, nine demonstrated successful Y90 delivery to the target lobe at ^90^Y PET/CT. MR volumetry demonstrated dose-dependent atrophy of the target lobe, with periportal fibrosis and dose-dependent decrease in hepatocyte proliferation seen at HE staining and Ki67 immunofluorescence histology.

Y90 microspheres are used for transarterial radioembolization (TARE) in a variety of liver tumors, the greatest amount of evidence obtained from studies in hepatocellular carcinoma (HCC) and metastatic colorectal cancer (mCRC). Besides its effects on tumor tissue, induction of parenchymal hypertrophy in non-treated areas following TARE has been studied by several groups following an initial case report published in 2009 [2]. Traditional Y90 TARE has somewhat drifted out of focus in recent years following the publication of large randomized trials in HCC and treatment-naïve mCRC (SARAH, SIRveNIB, SORAMIC, SIRFLOX, FOXFIRE, FOXFIRE Global), that have all failed to reach their primary endpoints of overall or progression-free survival. However, as subgroups potentially benefitting from TARE were identified in these studies and their failure has at least partly been attributed to inappropriate patient selection, the use of Y90 microspheres in the treatment of liver malignancies continues to be an area of great scientific interest. New research strategies are gradually emerging. Perhaps the most promising field for Y90 in the future is its use in “oligometastatic,” potentially curable patients in conjunction with local liver tumor treatments, i.e., surgery, thermal ablation, or the combination of both. A blinded post hoc analysis of imaging studies obtained in SIRFLOX has demonstrated that TARE significantly increases the technical resectability of colorectal liver metastases compared to chemotherapy alone [3].

The study by Gordon et al. adds another small piece of information to this complex picture, even though, for the moment, this study does not provide information on whether the observed atrophy in the treated lobe is associated with meaningful hypertrophy in untreated liver areas, nor is it clear whether the results obtained in rats are transferable to humans, as their different vascular anatomy may have an impact on hypertrophy development [4, 5]. Ultimately, the combination of direct and cytokine- and immune-mediated Y90 effects on tumor tissue, extratumoral hepatic parenchyma, extrahepatic tumor cells, and tumor microenvironment will determine patient benefit, and the sooner enough pieces of information like this one are put together to develop a randomized trial of Y90 in conjunction with local tumor eradication in appropriately selected patients, the better.
